# Visual area V5/hMT+ contributes to perception of tactile motion direction: a TMS study

**DOI:** 10.1038/srep40937

**Published:** 2017-01-20

**Authors:** Tomohiro Amemiya, Brianna Beck, Vincent Walsh, Hiroaki Gomi, Patrick Haggard

**Affiliations:** 1 Institute of Cognitive Neuroscience, University College London, Alexandra House, 17 Queen Square London, WC1N 3AZ, United Kingdom; 2 NTT Communication Science Laboratories, NTT Corporation, 3-1 Wakamiya, Morinosato, Atsugi-shi, Kanagawa, 243-0198, Japan

## Abstract

Human imaging studies have reported activations associated with tactile motion perception in visual motion area V5/hMT+, primary somatosensory cortex (SI) and posterior parietal cortex (PPC; Brodmann areas 7/40). However, such studies cannot establish whether these areas are causally involved in tactile motion perception. We delivered double-pulse transcranial magnetic stimulation (TMS) while moving a single tactile point across the fingertip, and used signal detection theory to quantify perceptual sensitivity to motion direction. TMS over both SI and V5/hMT+, but not the PPC site, significantly reduced tactile direction discrimination. Our results show that V5/hMT+ plays a causal role in tactile direction processing, and strengthen the case for V5/hMT+ serving multimodal motion perception. Further, our findings are consistent with a serial model of cortical tactile processing, in which higher-order perceptual processing depends upon information received from SI. By contrast, our results do not provide clear evidence that the PPC site we targeted (Brodmann areas 7/40) contributes to tactile direction perception.

The skin is the body’s largest receptor surface, and a boundary for body defence; the brain must compute how stimuli move across the skin to coordinate interceptive movements. In touch, as in vision, motion perception depends on spatiotemporal patterns within a two-dimensional sensory receptor array^1^, yet the neural mechanisms underlying the perception of such spatiotemporal tactile motion patterns are not fully understood.

Previous studies of tactile motion processing in primates focussed on primary somatosensory cortex (SI) and posterior parietal cortex (PPC). Single-unit studies in SI identified directionally-sensitive tactile neurons in areas 3b, 1 and 2^2^,^3^,^4^. Response properties of these neurons resembled human perceptual capabilities, suggesting a role in tactile motion perception. Neurons in classical multisensory association areas, notably ventral intraparietal area (VIP), also show sensitivity to both visual and tactile motion, usually in congruent directions^5^,^6^.

Human neuroimaging studies are less conclusive regarding the role of SI in tactile motion processing. Of the few studies that compared tactile motion perception to a static tactile control, some found increased SI activation during motion perception^7^,^8^,^9^, while others did not^10^,^11^. This contrast is important for linking activations specifically to tactile *motion*, rather than other dimensions of touch. A recent multivariate pattern analysis (MVPA) study identified encoding of tactile motion direction in SI^12^, but causal evidence that human SI is involved in tactile motion processing is still lacking.

Several human neuroimaging studies linked the intraparietal sulcus (IPS) and inferior parietal lobule (IPL) to tactile motion processing^8^,^11^,^12^,^13^,^14^,^15^,^16^,^17^. However, some of these studies did not include a control condition with static tactile input^13^,^14^,^15^,^16^. Others used dot matrix displays to produce *apparent* motion by changing spatial patterns of static dots^8^,^11^,^12^. Perceptual performance for some apparent motion stimuli thus may be limited by static form perception. Indeed, one study that independently varied static tactile pattern and apparent tactile motion reported IPL activation linked to pattern perception but not to motion perception per se^9^, whereas another study that used a static tactile control stimulus with a coherent spatial pattern found activation in the IPS in response to tactile motion^8^. Consequently, the role of the PPC in tactile motion perception remains unclear.

The varied results from previous studies may reflect the lack of a consensus stimulus for studying tactile motion perception. Many studies used either a rotating cylinder with a grid of raised dots^18^,^19^,^20^ or a Braille-like dot matrix display^8^,^9^,^12^,^21^,^22^. The rotating cylinder moves in a single direction, periodically stimulating an array of tactile receptors. The participant detects changes in speed, not direction of motion. Thus, the task could, in principle, be performed by processing either the rate or the temporal rhythm of action potentials in a single mechanoreceptive afferent, rather than motion across the skin.

In dot matrix displays, a set of pins indent the skin, exciting fast-adapting type I (FAI) afferents sensitive to dynamic skin deformations^23^,^24^. This creates the sensation of a discrete wave travelling in a specific direction. However, the dot matrix produces apparent, not real, motion. The whole matrix remains in constant contact with the skin, although the microgeometry at any one moment varies across receptive fields. Importantly, the dots do not move across the skin, so shear, friction and tangential forces are absent. Therefore, dot matrix stimuli may not activate slowly adapting type I (SAI) or type II (SAII) afferents^25^, which may play an important role in direction perception^26^.

Few studies of tactile motion perception have involved continuous movement of a single stimulus across the skin, leading to successive stimulation of *several* afferent fibres in a systematic spatial arrangement. Perceptual studies of “graphaesthesia”, often involving identifying letters or shapes drawn passively on the skin^27^, confirm that such stimuli can be accurately perceived, but stimulus control in these studies was often only approximate. In a non-human primate study, the experimenters moved a single-point tactile stimulus across the fingerpad of the monkey in cardinal directions, and found a population code in SI neurons that was strongly correlated with stimulus direction^4^. However, this study did not include any perceptual variables, nor did it investigate the resolution or threshold for representing differences in tactile motion direction. The human neural mechanisms for fine perception of single-point tactile motion stimuli have not been studied, to our knowledge.

Zangaladze and colleagues^28^ were the first to report that *visual* cortex contributes to spatial processing of static tactile stimuli. Since then, several human imaging studies have found that visual motion area V5/hMT+ responds to auditory motion^29^,^30^ and tactile motion^8^,^9^,^10^,^12^,^14^,^17^,^31^, suggesting a role for V5/hMT+ in multimodal motion processing (although there is at least one contradictory finding)^32^. To our knowledge, no single-unit studies have reported tactile motion coding in homologous brain areas in non-human primates. The human imaging results, however, do appear to reflect tactile motion processing in V5/hMT+ rather than related but epiphenomenal activity such as visual imagery. For example, tactile motion also activated V5/hMT+ in the congenitally blind^10^,^11^,^17^. Additionally, disrupting V5/hMT+ with repetitive transcranial magnetic stimulation (rTMS) impairs detection of changes in the speed of tactile texture flow^18^,^20^.

As discussed above, detecting changes in speed and detecting changes in direction are functionally distinct processes. In principle, tactile speed discrimination could be done solely based on the rate or rhythm of tactile stimulation at a single skin site, without any requirement to integrate information about changing spatial location on the skin. To truly demonstrate an involvement in tactile *motion* processing, one must use a task requiring the integration of both temporal and spatial information from the moving stimulus, such as direction discrimination. To date, no studies have directly tested whether V5/hMT+ plays a causal role in perceiving the *direction* of moving tactile stimuli.

Surprisingly, the most basic, natural stimulus for tactile motion perception—the motion of a single tactile point across the skin—has been largely neglected by modern human perceptual neuroscience. Here, we moved a single object, similar to the stimulus used in a previous non-human primate study^4^, across the human fingertip with controlled spatial and temporal trajectories. Participants judged whether motion direction deviated ‘toward’ or ‘away’ from the egocentre (Fig. 1). To investigate the neural mechanisms underlying the perception of this true motion stimulus, we disrupted activity in SI, PPC (Brodmann areas 7/40) and V5/hMT+ in the left hemisphere using online double-pulse TMS. Because these areas are relatively close together, and in some cases directly connected^33^,^34^, we could not make strong predictions about selective effects of stimulating one area compared to another nearby area. Rather, we compared each of the three active TMS conditions to a sham TMS condition using a rotated coil over the vertex, with appropriate correction for multiple comparisons. We predicted that disrupting both SI and V5/hMT+ would reduce tactile direction discrimination because previous studies reported directionally sensitive activity in both these areas^2^,^3^,^4^,^12^. Since the PPC may be more involved in perceiving static tactile patterns than tactile motion per se^9^,^35^, we predicted that PPC stimulation might not disrupt direction perception of our single-point tactile motion stimulus.

## Results

### Accuracy

Our data, expressed as percent correct scores, did not violate the assumptions of normality (Shapiro-Wilk tests, *p* ≥ 0.180) or sphericity (Mauchly’s test, *p* = 0.284). Additionally, the 95% confidence intervals were interpretable in all conditions (all lower bounds >0%, all upper bounds <100%). Since parametric assumptions were not violated, we analysed direction discrimination (percent correct) using a repeated measures ANOVA with the factor ‘TMS condition’ (SI, PPC, V5/hMT+ or rotated-coil sham). The effect of TMS was significant, *F*(3, 51) = 5.06, *p* = 0.0038, η^2^ = 0.073. Our predictions focussed on whether TMS at each active site influenced motion perception relative to sham TMS; therefore, our key inferences are based on comparisons between each active TMS condition and the sham TMS conditions. We used post-hoc comparisons with a Holm-Bonferroni correction, which appropriately adjusts the overall type I error rate. Compared to the sham condition (M = 76.1%, SD = 11.1%), participants were less accurate when TMS was applied over SI (M = 66.9%, SD = 14.0%; *t*(51) = −3.66, *p*_adj_ = 0.0036, Cohen’s d = 0.73) and over V5/hMT+ (M = 68.6%, SD = 12.0%; *t*(51) = −2.99, *p*_adj_ = 0.021, Cohen’s d = 0.65), but not over PPC (M = 70.7%, SD = 13.5%; *t*(51) = −2.16, *p*_adj_ = 0.140, Cohen’s d = 0.44) (Fig. 2a).

We performed further analyses to investigate whether participants may have indirectly judged motion direction based only on the end-points of each stimulus (Supplementary Information). The results of these analyses do not support the idea that participants adopted such a strategy.

### Signal detection analysis

TMS might influence either perceptual sensitivity (i.e., loss of information about the direction of motion) or post-perceptual response bias (i.e., tendency to perceive all stimuli as moving ‘toward’ or ‘away’ irrespective of the actual direction). Signal detection theory offers a framework for distinguishing sensitivity (d’) and bias (criterion) effects^36^. We hypothesized that areas causally involved in processing tactile motion should yield a change in perceptual sensitivity when disrupted. We had no specific predictions regarding response bias.

We arbitrarily defined the ‘away’ direction as the target to be detected. A repeated measures ANOVA (Shapiro-Wilk tests: *p* ≥ 0.199; Mauchly’s test: *p* = 0.660) revealed a significant effect of TMS on tactile direction processing (d’), *F*(3, 51) = 3.77, *p* = 0.016, η^2^ = 0.054. Holm-Bonferroni tests showed that, relative to the sham condition (M = 1.77, SD = 0.84), TMS over SI (M = 1.20; SD = 1.00; *t*(51) = −3.06, *p*_adj_ = 0.024, Cohen’s d = 0.62) and V5/hMT+ (M = 1.26, SD = 0.95; *t*(51) = −2.73, *p*_adj_ = 0.045, Cohen’s d = 0.57) reduced sensitivity (d’), while TMS over PPC did not (M = 1.40, SD = 1.04; *t*(51) = −2.03, *p*_adj_ = 0.192, Cohen’s d = 0.39) (Fig. 2b).

A repeated measures ANOVA (Shapiro-Wilk tests: *p* ≥ 0.372; Mauchly’s test: *p* = 0.792) also showed a significant effect of TMS on response bias (criterion), *F*(3, 51) = 2.84, *p* = 0.047, η^2^ = 0.048. Holm-Bonferroni post-hoc comparisons showed a non-significant trend of TMS over SI biasing participants in favour of ‘away’ responses (M = −0.11, SD = 0.58; *t*(51) = −2.58, *p*_adj_ = 0.078, Cohen’s d = 0.50). TMS over PPC (M = −0.02, SD = 0.47; *t*(51) = −1.66, *p*_adj_ = 0.412, Cohen’s d = 0.37) and V5/hMT+ (M = 0.12, SD = 0.45; *t*(51) = −0.34, *p*_adj_ = 0.732, Cohen’s d = 0.08) did not affect bias relative to the sham condition (M = 0.16, SD = 0.50; Fig. 2c).

## Discussion

We developed a tactile motion task in which participants judged the direction of a single tactile point moving across the fingertip. We applied double-pulse TMS to cortical areas SI, PPC and V5/hMT+, as well as sham TMS, shortly after onset of tactile motion. Both TMS over SI and TMS over V5/hMT+ disrupted direction discrimination relative to sham TMS. Conversely, TMS over PPC did not significantly affect performance. Signal detection analysis showed that these effects were due to reduced sensitivity, and were not merely changes in response bias. We conclude that both SI and, more intriguingly, V5/hMT+, contribute to perception of tactile motion direction. Our analyses were based on comparing each of the three stimulation sites to a sham condition, with appropriate correction for multiple comparisons, rather than searching for differences between any two stimulation sites. That is, we followed a classical neuropsychological logic of investigating whether each of several sites made a necessary contribution to tactile motion perception.

TMS can have both local and remote effects^37^. In particular, stimulating one area may also disrupt processing in areas connected with the stimulation site. A remote effect would mean, for example, that SI stimulation could affect processing in areas known to be connected to SI, such as PPC^33^,^34^. However, we did not find that stimulation targeted specifically at the PPC significantly interfered with tactile motion perception. This makes it unlikely that the effects of stimulating SI in fact reflect a remote effect mediated by PPC, since any such remote effect should be weaker than direct PPC stimulation, not stronger. Further, no direct connection has been identified between SI and V5/hMT+. Therefore, stimulation of SI is less likely to have disruptive effects on V5/hMT+ than on PPC.

We have stimulated three locations over the temporal and parietal lobes. Unavoidable passive spread of effects from the focus of stimulation to adjacent areas reduces the probability of finding differences between adjacent sites. We therefore preferred to investigate evidence for disruption at each site, relative to a sham TMS control condition. This design allows us to identify areas involved in tactile motion direction perception, but does not allow claims about selectivity, or about the *relative* importance of one area compared to another. Importantly, we do not claim a *selective* effect of TMS over V5/hMT+ or SI, relative to PPC. Rather, we conclude that V5/hMT+ and SI *both* contribute to direction perception for a tactile stimulus moving across the skin. In the case of PPC, the null hypothesis of no involvement in tactile motion perception could not be rejected. We must note, however, that the absence of a significant effect of PPC stimulation could also reflect a problem of statistical power. Further research will be necessary to resolve this issue.

A previous MVPA study^12^ identified neural patterns in V5/hMT+ that could classify highly discriminable (leftward vs. rightward) tactile motions. Our task required finer discrimination of tactile motion direction, since the variations in directions of motion were close to each individual’s discrimination threshold. Further, our use of TMS allows us to make causal inferences about the roles of the targeted brain areas in tactile direction processing. Our finding therefore extends previous work with direct causal evidence for a role of V5/hMT+ in acute perception of differences in the direction of a single point moving across the skin.

Our results contribute to the view of V5/hMT+ as a *multimodal* motion area rather than a purely visual area. Previous studies have found V5/hMT+ activity related to both auditory motion^29^,^30^ and tactile motion^8^,^9^,^10^,^12^,^14^,^17^,^31^ (but see contradictory findings)^32^. Additionally, this area responds to vestibular self-motion in both humans^38^ and monkeys^39^,^40^,^41^. In one view, V5/hMT+ might house visual imagery, yet not be multimodal^42^,^43^. For example, tactile motion might be transformed into a visual code. However, intermodal transformations would predict a common code for visual and tactile directions, yet one study found that visual and tactile MVPA patterns for a given motion direction were not shared^12^. Furthermore, in sighted individuals, the anterior portion of V5/hMT+ tends to be activated by motion in the tactile modality, whereas the posterior portion appears specific to visual motion processing^10^,^17^,^44^. These findings suggest the involvement of V5/hMT+ in tactile motion processing cannot be fully explained by visual imagery. Instead, V5/hMT+ may contain distinct subpopulations of neurons that process motion from different sensory modalities.

We also found disruption of tactile direction discrimination when TMS was applied over SI. This could reflect disruption of direction-sensitive tactile neurons in SI^2^,^3^,^4^. Alternatively, applying TMS over SI may have impaired direction discrimination by disrupting a common early tactile processing stage. For example, if SI stimulation simply masked information about the tactile stimulus, then a putative higher-order motion processing area would receive degraded input from SI. This possibility is consistent with classical models of a serial somatosensory processing pathway^45^,^46^. Our results cannot conclusively distinguish whether TMS over SI disrupts tactile motion processing within SI, or merely disrupts inputs to tactile motion processing housed at subsequent stages in a hierarchical pathway. In a control task, we confirmed that stimulation at our SI location significantly reduced simple detection of electrical stimuli (Supplementary Fig. S1). Effects of SI stimulation on motion perception due to disrupted early processing cannot, therefore, be ruled out. However, our data are also consistent with TMS disrupting two intermixed neural populations within SI, one underlying tactile detection, and the other underlying direction-selective processing.

Unlike SI and V5/hMT+ TMS, TMS over PPC (Brodmann areas 7/40) did not significantly impair tactile motion perception. Clearly, this null result could reflect factors such as low statistical power, or differences in effectiveness of stimulation. Therefore, we cannot completely exclude some contribution of PPC to tactile motion perception. However, our data remain consistent with the null hypothesis that PPC is not involved in processing the direction of tactile motion. Interestingly, several neuroimaging studies reported IPS/IPL activations in response to tactile motion, suggesting that our PPC stimulation should have been effective^8^,^11^,^12^,^13^,^14^,^15^,^16^,^17^. Again, we believe that careful consideration of the stimulation parameters for tactile motion may resolve the apparent controversy between those imaging results and ours. Our stimulus involved motion of a single tactile point across the skin, while those studies typically used a Braille-like dot array, providing spatial patterns of indentation. We speculate that the activation of PPC by apparent tactile motion in dot matrix studies might be linked to processing spatially extended patterns rather than to processing tactile motion direction per se. This view is supported by other studies showing PPC activation during purely static tactile spatial pattern processing^9^,^35^. However, we must also consider that the PPC is a large, functionally organised cortical area, and we targeted only one location within this area (Brodmann areas 7/40, within the IPL)^47^. It remains possible that other parts of the PPC may be involved in processing tactile motion direction.

In conclusion, we have investigated the neural mechanisms for perceiving the direction of a tactile point moving across the skin. We report what we believe to be the first causal study of motion processing for this basic class of stimulus. Our results confirm that V5/hMT+ underlies perception of tactile motion direction, and also shed new light on the organisation of cortical somatosensory processing pathways.

## Methods

### Participants

A required sample size of 18 participants was estimated using G*Power 3.1^48^, based on a desired power of 0.95 and the average effect size of impaired tactile motion processing caused by TMS over V5/hMT+ in previous studies (η^2^ = 0.53)^18^,^20^. Eighteen healthy volunteers (right-handed; 9 females; 18–43 years old) participated for payment. They were screened for contraindications to TMS^49^,^50^. All volunteers gave written informed consent to participate in the experiment, and all experimental protocols were approved by the University College London Research Ethics Committee. The experiment was carried out in accordance with the guidelines in the Code of Ethics of the World Medical Association (Declaration of Helsinki). All participants were naïve about the aim of the study.

### Apparatus

**Figure 1:**
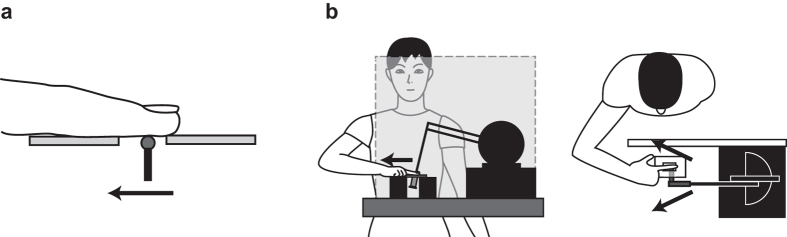
Illustrations of the experimental procedure. (**a**) Illustration of tactile motion stimulus. (**b**) Illustration of experimental apparatus.

**Figure 2:**
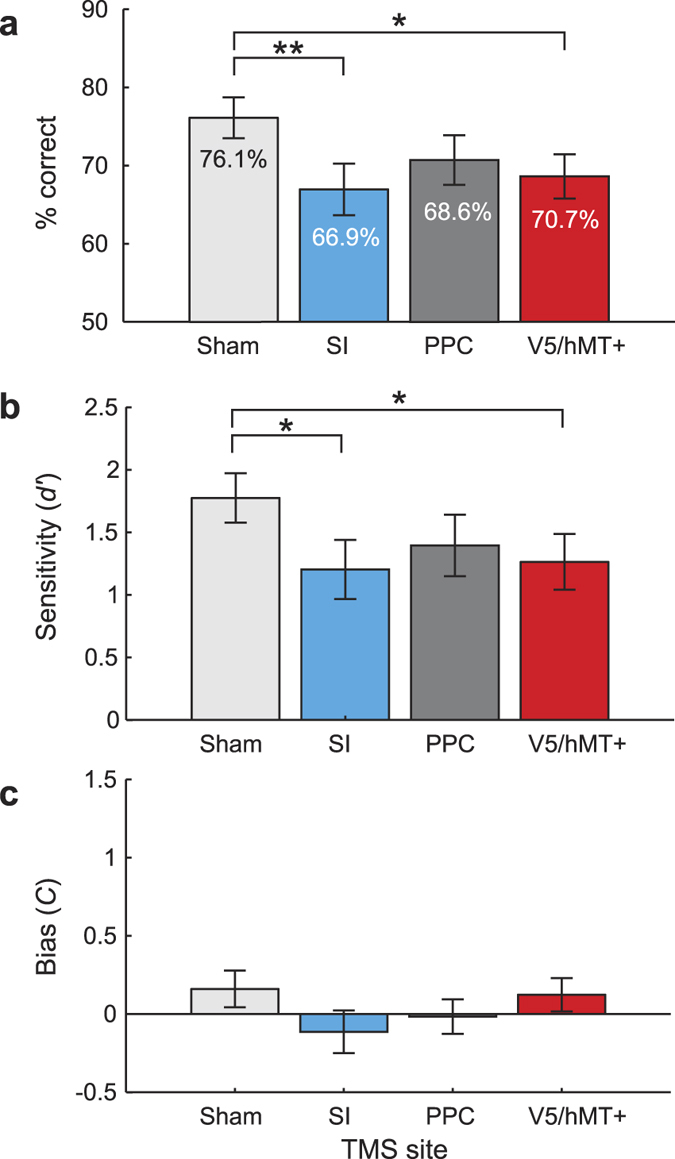
Mean (±SEM, N = 18) tactile direction discrimination performance in terms of (**a**) percentage correct, (**b**) sensitivity (d’) and (**c**) response bias (criterion). *p < 0.05, **p < 0.01.

A spherical probe (4 mm dia) attached to an L-shaped extension arm was moved across the fingerpad using a 3-dimensional force feedback device (PHANToM Premium 1.0, Geomagic Inc., USA) (Fig. 1a). A height-adjustable plastic plate with a rectangular gap was used to guide finger placement and to keep the probe in stable contact with the fingertip. The probe was located underneath the plate and moved upwards to swipe across the fingertip through the gap.

### Tactile motion stimulation

The right hand rested in a fixed position with the index finger placed over the gap and pointing leftward. The probe moved in a straight, distal-to-proximal line along the distal fingerpad with a deviation either toward or away from the egocentre. The velocity of the stimulus was 69 mm/s, with smoothly connected rising and falling phases lasting 30 ms each. This velocity was chosen because it generated a clear direction sensation in pilot tests and was within the range used in active surface exploration^51^,^52^ and in passive speed discrimination studies^19^. The amplitude of the two deviations angled ‘toward’ or ‘away’ was individually selected for each participant to match difficulty (see Procedure). Each tactile motion stimulus lasted 120 ms. The distance of stimulus travel (path length) on the fingerpad was approximately 6.2 mm.

At the beginning of each trial, the probe made static contact with the fingertip for 1 s. The initial position of the probe on the fingertip was jittered across trials (−2.0, 0.0, or 2.0 mm from the centre of the fingerpad) to prevent participants from judging direction by the final position of the probe only. At the end of the falling phase, the probe was immediately retracted from the skin.

### TMS

A MagStim Rapid^2^ magnetic stimulator (Magstim Co. Ltd., UK) with a figure-of-eight coil (each wing 70 mm dia) was used to deliver focal cortical stimulation. Target cortical sites—left SI, PPC and V5/hMT+ —were localised prior to the main experiment, according to established procedures^53^,^54^,^55^,^56^ (see Supplementary Information). We delivered double-pulse TMS 20 ms after the onset of tactile motion across the skin with an interpulse interval of 60 ms. This latency was chosen based on the most effective timing for disrupting tactile detection with TMS over SI in previous studies^57^,^58^. A 60-ms interval was used to ensure that disruption lasted for the duration of the tactile motion stimulus, considering that the effect of a single pulse of TMS lasts 50–150 ms^59^,^60^. TMS intensity was set to 60% of maximum stimulator output over all the sites.

### Procedure

Participants placed the right index finger over the gap. The experimenter adjusted the contact depth of the probe from the skin surface to be about 2–3 mm when moving across the finger. The right hand and the device were occluded so that direction could not be judged by sight (Fig. 1b). To take advantage of the apparent bias for centrifugal motion in V5/hMT+^61^, the participant’s right arm was pointed left. In this way, the tactile motion stimulus moved rightward down the long axis of the participant’s finger and away from the body midline.

The participants’ task was to discriminate whether the tactile motion stimulus deviated ‘toward’ or ‘away’ from their egocentre. Participants made unspeeded key presses with the left hand to respond. No feedback was given.

Each participant’s direction discrimination threshold was determined prior to the main experiment using a 1-up 3-down staircase method. The threshold was confirmed by running a practice session without TMS. If participants gave correct responses on less than 65% or more than 85% of the practice trials, the deviation angles were modified and the process was repeated until a reliable discrimination threshold was found. The mean angular difference at threshold was 24.28 ± 13.96 deg.

The experimental session consisted of eight blocks of 20 trials (two blocks per TMS condition), each lasting about 2 min. There were a total of 40 trials per TMS condition. Each trial lasted at least 5 s to limit any TMS carry-over effects. The order of TMS conditions was counterbalanced across participants using a modified Latin Square, and reversed using an ABCDDCBA design to minimise time-dependent effects. A break of at least 3 min was given between blocks to re-position the TMS coil and reduce effects of fatigue and sensory adaptation.

At the end of the experiment, participants completed the tactile detection SI localiser task as described in the Supplementary Information.

## Additional Information

**How to cite this article**: Amemiya, T. *et al*. Visual area V5/hMT+ contributes to perception of tactile motion direction: a TMS study. *Sci. Rep.* **7**, 40937; doi: 10.1038/srep40937 (2017).

**Publisher's note:** Springer Nature remains neutral with regard to jurisdictional claims in published maps and institutional affiliations.

## Supplementary Material

Supplementary Information
