# Supplementary material for: Visual area V5/hMT+ contributes to perception of tactile motion direction: a TMS study
Source: Sci Rep. 2017 Jan 20;7:40937. doi: 10.1038/srep40937 (PMC5247673; doi:10.1038/srep40937)
Supplement: Supplementary Information [file srep40937-s1.doc]

Visual area V5/hMT+ contributes to perception of tactile motion direction: a TMS study

Tomohiro Amemiya^1,2,3^, Brianna Beck^1,3^, Vincent Walsh^1^, Hiroaki Gomi^2,4^, Patrick Haggard^1,4^

^1^Institute of Cognitive Neuroscience, University College London, Alexandra House, 17 Queen Square London, United Kingdom WC1N 3AZ

^2^NTT Communication Science Laboratories, NTT Corporation, 3-1 Wakamiya, Morinosato, Atsugi-shi, Kanagawa, Japan 243-0198

^3^Co-first author

^4^Co-senior author

Corresponding author:

Patrick Haggard

Tel.: +44 (0)207 679 1153

Fax: +44 (0)207 813 2835

E-mail: p.haggard@ucl.ac.uk

**Supplementary Information**

**Supplementary Methods**

### *Localisation of primary somatosensory cortex (SI)*

The hand motor area of the left motor cortex was located in each participant using single pulses of transcranial magnetic stimulation (TMS). The participant sat in a relaxed position with the thumb and index finger of the right hand pinched together. The TMS coil was first placed 5 cm lateral and 1 cm anterior to the vertex. TMS intensity was increased from 30% to 65% of maximum intensity in steps of 5% until a visible motor twitch was found, according to both the participant’s report and the experimenters’ observations. The coil was then moved in 1 cm steps in anterioposterior and mediolateral directions until the site for evoking the largest twitches was found. The hand motor area was successfully found in this manner for 16 participants. For the remaining 2 participants, this procedure did not produce any twitches. Therefore, we considered 5 cm lateral and 1 cm anterior to the vertex as a plausible estimate for the location of the hand motor area in these participants^1^.

SI was located by moving the coil 2 cm posteriorly from the hand motor area, following a method used in previous TMS studies targeting SI^2,3^. This position has been shown to overlie the postcentral gyrus^4^. The mean position of SI was 4.94 ± 0.42 cm lateral and 1.06 ± 0.94 cm posterior to the vertex. During SI stimulation, the coil was held with the handle pointing backwards and parallel to the midline. A double-pulse of TMS was delivered over this spot to ensure that no detectable motor twitches were produced.

Furthermore, a tactile detection task was used to confirm the localisation of SI. Transcutaneous electrical stimulation was presented through stainless steel ring electrodes affixed to the right index finger. Participants reported whether they felt any tactile sensation on the finger. A 1-up 3-down staircase procedure was conducted to find the electrical stimulation level that yielded 79.4% correct detection in each participant. The session proceeded for 8 reversals, up to a maximum of 120 trials. Initial current intensity was 0.5 mA. The step size started at 0.1 mA and reduced to 0.05 mA after two reversals. The threshold was calculated by discarding the first two reversals and averaging the remaining reversals. The mean current intensity at threshold (± SD) was 0.76 ± 0.37 mA.

The tactile detection task consisted of four blocks (two blocks of SI stimulation and two blocks of sham stimulation). Each block contained 20 trials and lasted about 2 min. There were thus a total of 40 trials for each TMS condition. The order of TMS conditions was counterbalanced across participants, and reversed using an ABBA design to minimize time-dependent effects. Half of the trials in each block contained a near-threshold electrical stimulus, and the other half did not.

Single pulses of TMS at 60% of the maximum stimulator output were delivered 20 ms after the onset of the electrotactile stimulus, either over SI (active stimulation)—at which time it should disrupt tactile detection^5^—or over the vertex with the coil rotated 90° in the vertical plane (sham stimulation). The results showed that sensitivity (d’) was significantly different between sham and SI TMS conditions, t(17)=3.09, p=.0033 (Supplementary Fig. S1a). No difference in response bias was observed, t(17)=-1.36, p=0.90 (Supplementary Fig. S1b). These data provide evidence that the chosen location 2 cm posterior to the hand motor area was indeed over the hand somatosensory area. The two participants for whom the hand motor area was not functionally localised also exhibited reduced sensitivity when TMS was applied over SI (2.49 vs 2.12 for one and 2.93 vs 2.12 for the other; d’ under sham vs d’ under SI). This trend was similar to that observed in the other participants whose hand motor areas were functionally localised, providing evidence that SI was also properly targeted in these two participants.


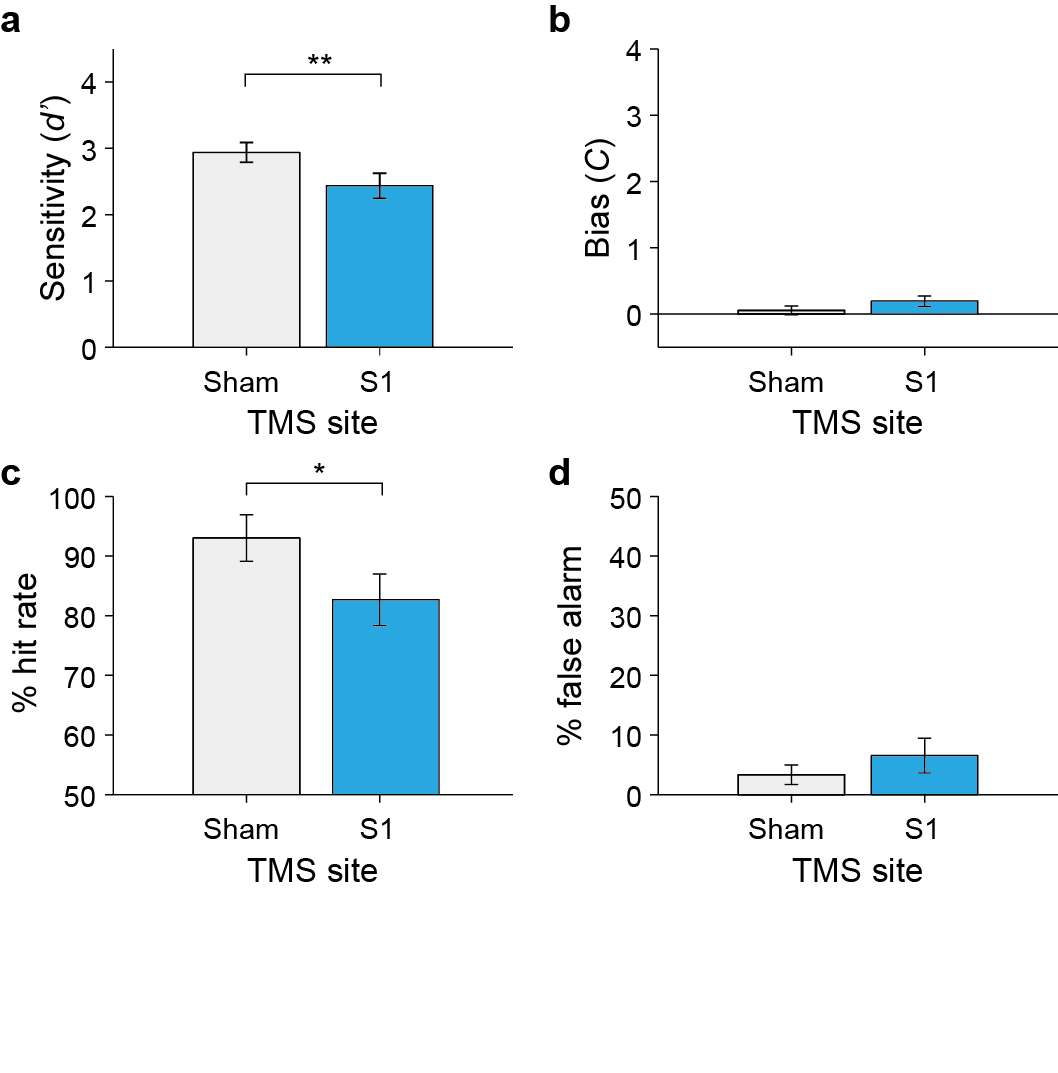


Supplementary Figure S1. Mean (± SEM, N = 18) tactile detection performance in terms of **a**) sensitivity (d’), **b**) response bias (criterion), **c**) hit rate, and **d**) false alarm rate. **p* < .05, ***p* < .01.

### *Localisation of middle temporal complex (V5/hMT+)*

We located left V5/hMT+ using the functional method of TMS-induced phosphenes, which has been used in a number of studies on V5/hMT+ function^6,7^. The TMS coil was initially placed 3 cm dorsal and 5 cm lateral to the inion, and then moved in steps of 1 cm in dorsoventral and mediolateral directions until the spot was found over which double-pulses of TMS elicited the strongest moving phosphenes. All 18 blindfolded participants reported seeing phosphenes. (Nine of them reported distinct impressions of motion, while the other nine reported bright spots or flashes). While moving phosphenes are considered the gold standard for functional localisation of V5/hMT+, it is common for some participants to experience only static phosphenes with V5/hMT+ stimulation, even when individual MRI-guided neuronavigation is used for localisation^8^. The mean position of V5/hMT+ was 3.64 ± 0.87 cm dorsal and 5.06 ± 1.21 cm lateral to the inion, which is in accordance with the coordinates reported in studies that used fMRI-guided neuronavigation to localise V5/hMT+^9,10^.

### *Localisation of posterior parietal cortex (PPC) and vertex*

The left PPC and the vertex were marked on the basis of scalp measurements for all participants.

For the left PPC, a site 9 cm dorsal and 6 cm lateral to the inion was used. This is approximately the location of P3 in the international 10–20 system, which overlies Brodmann areas 7/40 on the inferior parietal lobe and the intraparietal sulcus^11^.

The position of the vertex was identical to Cz in the international 10–20 system, i.e., the midpoint of the nasion, inion, and preauricular points. In the sham vertex TMS condition, the TMS coil was rotated 90° in the vertical plane, such that the magnetic field did not pass through the skull. The sham TMS condition thus controlled for nonspecific effects of TMS such as auditory and sensory artefacts.

**Supplementary Results**

### *Indirect discrimination of motion direction based on stimulus end-points?*

In the experiment, the initial points of the tactile motion stimulus were jittered from -2 to 2 mm. However, the end-points of the stimulus were also jittered from -4.6 to 4.6 mm. One may speculate that stimuli with end-points far from the finger centre would be perceived more accurately than trials with end-points close to the finger centre, if end-point location were used as a cue. We further analysed discrimination performance of tactile motion direction (% correct) for trials with end-points at varying distances from the centre of the fingerpad using a two-way repeated measures ANOVA with the factors of end-point distance (±4.6 mm, ±2.6 mm or ±0.6 mm) and TMS site (SI, PPC, hMT+ or sham). There was a main effect of TMS site, *F*(3,51) = 6.14, *p* = .001, η_p_^2^ = .265, but neither a main effect or end-point distance, *F*(2,34) = 0.54, *p* = .587, η_p_^2^ = .031, nor a TMS site x end-point distance interaction, *F*(6,102) = 0.93, *p* = .475, η_p_^2^ = .052. In fact, performance in the sham TMS condition was non-significantly but numerically better on trials ending near the finger centre (±0.6 mm), relative to trials ending far from the finger centre (±4.6 mm; Supplementary Fig. S2). This analysis suggests that participants did not use end-points as a cue to indirectly discriminate tactile motion direction.


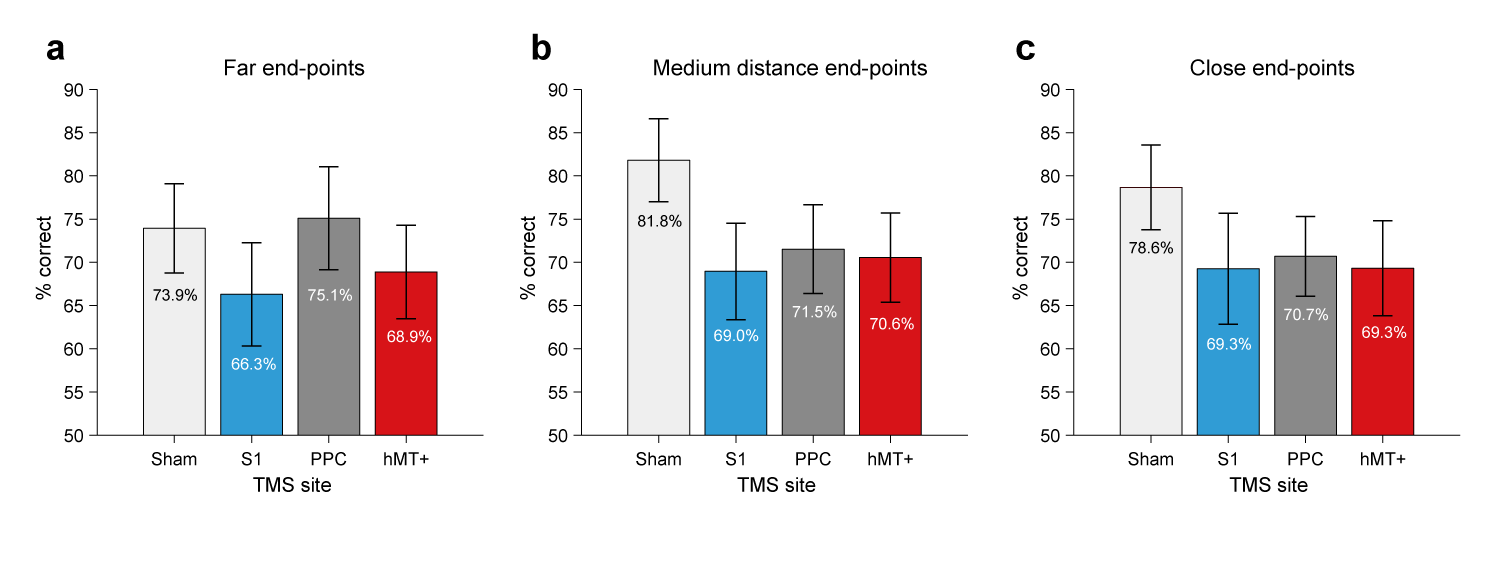


Supplementary Figure S2. Performance on trials with end-points **a**) far from the centre of the fingerpad (±4.6 mm), **b**) at an intermediate distance from the centre of the fingerpad (±2.6 mm), and **c**) near the centre of the fingerpad (±0.6 mm).

**Supplementary References**

1 Classen, J. *et al.* Multimodal output mapping of human central motor representation on different spatial scales. *J. Physiol. (Lond.)* **512**, 163-179, doi:10.1111/j.1469-7793.1998.163bf.x (1998).

2 Tamura, Y. *et al.* Disordered plasticity in the primary somatosensory cortex in focal hand dystonia. *Brain* **132**, 749-755, doi:10.1093/brain/awn348 (2009).

3 Wolters, A. *et al.* Timing-dependent plasticity in human primary somatosensory cortex. *J. Physiol. (Lond.)* **565**, 1039-1052, doi:10.1113/jphysiol.2005.084954 (2005).

4 Okamoto, M. *et al.* Three-dimensional probabilistic anatomical cranio-cerebral correlation via the international 10-20 system oriented for transcranial functional brain mapping. *Neuroimage* **21**, 99-111, doi:10.1111/j.1469-7793.1998.163bf.x (2004).

5 Cohen, L. G., Bandinelli, S., Sato, S., Kufta, C. & Hallett, M. Attenuation in detection of somatosensory stimuli by transcranial magnetic stimulation. *Electroencephalogr. Clin. Neurophysiol.* **81**, 366-376, doi:10.1016/0168-5597(91)90026-T (1991).

6 Cowey, A. & Walsh, V. Magnetically induced phosphenes in sighted, blind and blindsighted observers. *Neuroreport* **11**, 3269-3273, doi:10.1097/00001756-200009280-00044 (2000).

7 Pascual-Leone, A. & Walsh, V. Fast backprojections from the motion to the primary visual area necessary for visual awareness. *Science* **292**, 510-512, doi:10.1126/science.1057099 (2001).

8 Murd, C., Einberg, A. & Bachmann, T. (2012). Repetitive TMS over V5/MT shortens the duration of spatially localized motion aftereffect: the effects of pulse intensity and stimulation hemisphere. *Vision Res.* **68**, 59-64, doi:10.1016/j.visres.2012.07.009 (2012).

9 Sack, A. T., Kohler, A., Linden, D. E., Goebel, R. & Muckli, L. The temporal characteristics of motion processing in hMT/V5+: combining fMRI and neuronavigated TMS. *NeuroImage* **29**, 1326-1335, doi:10.1016/j.neuroimage.2005.08.027 (2006).

10 Vetter, P., Grosbras, M. H. & Muckli, L. TMS over V5 disrupts motion prediction. *Cereb. Cortex* **25**, 1052-1059, doi:10.1093/cercor/bht297 (2015).

11 Herwig, U., Satrapi, P. & Schonfeldt-Lecuona, C. Using the international 10-20 EEG system for positioning of transcranial magnetic stimulation. *Brain Topogr.* **16**, 95-99, doi:10.1023/B:BRAT.0000006333.93597.9d (2003).
